# Trends and regional variations of gonococcal antimicrobial resistance in the Netherlands, 2013 to 2019

**DOI:** 10.2807/1560-7917.ES.2022.27.34.2200081

**Published:** 2022-08-25

**Authors:** Maartje Visser, Hannelore M Götz, Alje P van Dam, Birgit HB van Benthem

**Affiliations:** 1 Centre for Infectious Disease Control, National Institute for Public Health and the Environment, Bilthoven, the Netherlands; 2 Department Infectious Disease Control, Municipal Public Health Service Rotterdam-Rijnmond (GGD Rotterdam), Rotterdam, the Netherlands; 3 National Reference Laboratory for Neisseria gonorrhoeae, Public Health Laboratory, Amsterdam Health Service, Amsterdam, the Netherlands

**Keywords:** Neisseria gonorrhoeae, antimicrobial resistance, surveillance, The Netherlands

## Abstract

**Background:**

Gonococcal antimicrobial resistance is emerging worldwide and is monitored in the Netherlands in 18 of 24 Sexual Health Centres (SHC).

**Aim:**

To report trends, predictors and regional variation of gonococcal azithromycin resistance (AZI-R, minimum inhibitory concentration (MIC) > 1 mg/L) and ceftriaxone decreased susceptibility (CEF-DS, MIC > 0.032 mg/L) in 2013–2019.

**Methods:**

SHC reported data on individual characteristics, sexually transmitted infection diagnoses, and susceptibility testing (MIC, measured by Etest). We used multilevel logistic regression analysis to identify AZI-R/CEF-DS predictors, correcting for SHC region. Population differences’ effect on regional variance of AZI-R and CEF-DS was assessed with a separate multilevel model.

**Results:**

The study included 13,172 isolates, predominantly (n = 9,751; 74%) from men who have sex with men (MSM). Between 2013 and 2019, annual proportions of AZI-R isolates appeared to increase from 2.8% (37/1,304) to 9.3% (210/2,264), while those of CEF-DS seemed to decrease from 7.0% (91/1,306) to 2.9% (65/2,276). Among SHC regions, 0.0‒16.9% isolates were AZI-R and 0.0−7.0% CEF-DS; population characteristics could not explain regional variance. Pharyngeal strain origin and consultation year were significantly associated with AZI-R and CEF-DS for MSM, women, and heterosexual men. Among women and heterosexual men ≥ 4 partners was associated with CEF-DS, and ≥ 10 with AZI-R.

**Conclusions:**

No resistance or decreasing susceptibility was found for CEF, the first line gonorrhoea treatment in the Netherlands. Similar to trends worldwide, AZI-R appeared to increase. Regional differences between SHC support nationwide surveillance with regional-level reporting. The increased risk of resistance/decreased susceptibility in pharyngeal strains underlines the importance of including extragenital infections in gonococcal resistance surveillance.

## Introduction

*Neisseria gonorrhoeae* infection (gonorrhoea) is one of the most common sexually transmitted infections (STI) in the Netherlands, with more than 19,500 diagnosed cases estimated in 2019 [1]. *N. gonorrhoeae* is known to have developed antimicrobial resistance (AMR) against many antibiotics, threatening the availability of effective treatment [2]. In the Netherlands, ceftriaxone has been used as monotherapy for gonorrhoea since 2006 [3]. While resistance to azithromycin has been increasing in the country since 2012, resistance to ceftriaxone has not yet been reported [1]. It has, however, been reported by other countries, including cases of gonorrhoea resistant to both azithromycin and ceftriaxone, which are together the recommended first-line dual therapy for gonorrhoea infections in the World Health Organization (WHO) and European Centre for Disease Prevention and Control (ECDC) guidelines [4-7].

To monitor gonorrhoea AMR in the Netherlands, the Gonococcal Resistance to Antibiotics Surveillance (GRAS) was established in 2006. GRAS is a sentinel surveillance system with broad coverage, including a majority of Dutch Sexual Health Centres (SHC) and their affiliated laboratories. These SHC offer free STI testing and care for certain target groups who are considered at high risk for STI, such as people with STI symptoms, people who received partner notification, individuals below 25 years of age, and men who have sex with men (MSM) [1]. For all gonorrhoea patients diagnosed at SHC participating in GRAS, culture and susceptibility testing should be performed for azithromycin, cefotaxime, ceftriaxone, and ciprofloxacin. Results from the GRAS surveillance are then collected at the National Institute for Public Health and the Environment (RIVM), and are used to inform treatment and prevention guidelines, and provide detailed insights in AMR of *N. gonorrhoeae* in the Netherlands [8]. In this study, the gonococcal AMR trends in the Netherlands are analysed using GRAS data on azithromycin and ceftriaxone from 2013 to 2019. Until now, GRAS results have only been presented on a national level. However, data collection and susceptibility testing in GRAS are executed by regional SHC and laboratories, so there may be regional variations in the outcomes of the GRAS surveillance. Therefore, in addition to describing national trends, this study also explores these regional differences.

## Methods

### Data collection

This study uses national SHC and GRAS surveillance data, which are routinely collected via a web-based application by the RIVM. The SHC surveillance data comprise pseudonymised information on individuals’ characteristics, sexual behaviour, and STI testing and diagnoses from each person at each consultation. For SHC participating in GRAS, these data also include culture and susceptibility testing results for patients with gonorrhoea, if available. At the SHC, nucleic acid amplification tests (NAATs) are used for gonorrhoea diagnosis. Heterosexual men are tested at the urogenital location only. For women, additional pharyngeal and anorectal tests are performed on indication. Anorectal testing is indicated by reported exposure although more extensively in some SHC. Pharyngeal testing is not mandatory for women other than sex workers reporting oral exposure, and testing rates also differ per SHC. Women reporting sex work as well as MSM are tested on all three anatomical locations.

SHC participating in GRAS send a sample for culture to the laboratory from all individuals with gonorrhoea symptoms or people who tested positive for gonorrhoea with NAAT. If the culture is positive as well, susceptibility testing is performed for azithromycin, cefotaxime, ceftriaxone and ciprofloxacin using Etest. The brand of Etest and culture plates that are used differ between participating laboratories. Minimum inhibitory concentration (MIC) values are then reported to the RIVM. As this study uses routinely collected pseudonymised surveillance data, ethical approval and informed consent were not required by Dutch law. However, due to the implementation of the General Data Protection Regulation in 2018, all SHC-attendees were asked for consent to share their data with the RIVM from May 2018 onwards, resulting in ca 15% of consultations in 2018 and 2019 not being reported. The percentage non-consenting attendees was the same (15%) among heterosexual men and women and MSM and among attendees under and over 25 years of age [1].

### Study population and definitions

All gonorrhoea patients, diagnosed by NAAT or culture, who had a consultation between 2013 and 2019 at SHCs participating in GRAS were included in the study. Between 2013 and 2019, 18 of 24 Dutch SHC participated in GRAS. However, four SHC located in certain regions (Supplementary Figure S1) did not participate for the whole study period: regions 6 and 10 started in 2015, region 7 in 2016, and region 5 stopped in 2019. Region 18 included only a total of three strains and was excluded.

Between 2013 and 2015 only one MIC value per antibiotic per patient could be reported. From 2016 onwards this was possible for multiple anatomical locations per patient. Therefore, if after 2016 susceptibility results from multiple anatomical locations within one patient were available, only one was included in these analyses. We included the isolate with the highest MIC-value, and in case of equal MICs, isolates were included according to an order of preference based on anatomical location: pharyngeal, rectal, urogenital (urethral/vaginal/cervical). This is the same practice as SHC used before 2016 to decide which MIC values to report.

Azithromycin resistance was defined as MIC > 1 mg/L, based on the European Committee on Antimicrobial Susceptibility Testing (EUCAST) MIC epidemiological cut off (ECOFF) [9]. For ceftriaxone no resistance has been reported yet in the Netherlands, so reduced susceptibility was used as outcome in the analyses, defined as a MIC above 0.032 mg/L, which is the ECOFF as defined by EUCAST [10].

Year of consultation was included in the regression analyses to see if trends over time remained significant after correction for other variables. Also, in 2015, triaging at the SHC became more strict, causing the population being tested at the SHC to shift towards higher risk groups. Additionally, before 2015, heterosexuals < 25 years old with no risk factors were only tested for chlamydia, and after 2015 for both chlamydia and gonorrhoea. Due to low numbers, years could not be included separately, and a categorical variable was used.

Sex and sexual orientation were based on sex and self-reported sexual behaviour. All women were grouped together, regardless of the sex of their sexual partners. Men were divided in heterosexual men and MSM (both homosexual and bisexual). Consultations from transgender clients were excluded (n = 66). 

Migration background from an STI/HIV endemic area was defined as being a first- or second-generation migrant from certain countries in Eastern Europe, Asia, Africa, or South America [11]. This is the same definition that is used in triage at the SHC. No further distinction was made in country of origin due to low numbers.

STI testing rate per region was calculated as the number of individuals aged 15–65 years with at least one test at the SHC per 1,000 inhabitants aged 15–65 years, using 2019 SHC and population census data. STI testing rate was included in analyses in the categories low (0.0–6.9 per 1,000) middle (7.0–12.9 per 1,000) and high (> 13.0 per 1,000). All other variables included in the analyses (STI symptoms, partner notification for STI, STI in the past year, number of partners in the past 6 months, HIV status) were self-reported by the SHC attendees.

### Data analyses

Descriptive analyses were used to assess GRAS participation per region and characteristics of the study population. To describe AMR evolution during the study period and MIC shift, the percentage of isolates with resistance or decreased susceptibility, MIC_50_, MIC_90_ and geometric mean MIC values were calculated over time at national level. To describe regional differences, the percentage of isolates with resistance or decreased susceptibility was also calculated for each individual SHC, including 95% confidence intervals (CI).

To assess predictors of AMR (for azithromycin) and decreased susceptibility (for ceftriaxone), multilevel logistic regression analyses were used. First, variables were assessed in univariable logistic regression. If significant (p < 0.1), the variables were included in the multivariable model. The final model was established using backward selection removing non-significant variables (p < 0.05). A random intercept for SHC region was used to correct for regional differences. Since the number of missing values were low, we used complete case analyses.

Second, another multilevel logistic regression model was built to see whether regional variance in AMR/decreased susceptibility could be explained by the population characteristics of the different regions. First, an ‘empty’ model was estimated, with resistance/decreased susceptibility as outcome and including no predictor variables but including a random intercept for region. This model thus shows the regional variance in AMR/decreased susceptibility. Then, population characteristics variables were added to the model. These were all the individual level variables that were also included in the regression model looking for predictors of resistance. We then calculated the proportional change in variance (PCV) of this ‘full’ model compared with the empty model to assess to what extend the regional variance could be explained by the added variables [12]. PCV = $\frac{V_{0}-V_{1}}{V_{0}}$ , where V_0_ is the regional variance in the empty model and V_1_ the regional variance in the model including all characteristics. To assess the contribution to regional variance of each separate variable we calculated the percentage of contribution per characteristic if it was removed from the extended model. Per cent (%) contribution = $\frac{V_{\left(1-k\right)}-V_{1}}{V_{\left(1-k\right)}}$ , where V_(1-k)_ is the regional variance in the model with one characteristic (k) removed.

## Results

### Characteristics of consultations with a positive culture for gonorrhoea

Between 2013 and 2019, the SHC participating in GRAS diagnosed 34,263 cases of gonorrhoea of which 13,172 (38.4%) included susceptibility testing results (consultations with positive cultures included in GRAS). Of all consultations with positive cultures, the majority (n = 9,617; 73.0%) was carried out in the two largest SHC regions (1 and 2). Most included consultations were from MSM (74.0%), persons older than 25 years (69.9%), and persons with no migration background from an STI/HIV endemic area (62.0%). Characteristics of consultations with positive cultures (included in GRAS) and consultations with lacking or negative cultures (not included in GRAS) were often significantly different (Table 1). The percentage of consultations with positive culture was higher among heterosexual men (49.8%), persons with an STI/HIV endemic migration background (43.6%), HIV-positive individuals (44.3%), persons who had a previous STI (42.8%) or reported STI symptoms (48.0%). There were relatively less persons who received partner notification (34.0%) (Table 1). Additional information on geographical location, population size and STI testing rates per region is given in Supplementary Table S1 and Supplementary Figure S1.

**Table 1:** Characteristics of consultations with gonorrhoea diagnoses and consultations including antimicrobial susceptibility testing results at Dutch Sexual Health Centres participating in GRAS, the Netherlands, 2013–2019 (n = 34,263 diagnoses)

Characteristics	Number of gonorrhoea diagnoses	Number of consultations for gonorrhoea with antimicrobial susceptibility testing results^a^	Proportion with antimicrobial susceptibility testing results (%) among total with such results	Proportion (%) of consultations with antimicrobial susceptibility testing results among gonorrhoea diagnoses	p-value
Total	34,263	13,172	100	38.4	
**SHC region**					
1	13,632	6,815	51.7	50.0	< 0.001
2	5,797	2,802	21.3	48.3	
3	3,322	1,103	8.4	33.2	
4	1,652	484	3.7	29.3	
5	806	364	2.8	45.2	
6	1,125	302	2.3	26.8	
7	880	280	2.1	31.8	
8	940	256	1.9	27.2	
9	1,648	199	1.5	12.1	
10	643	176	1.3	27.4	
11	592	162	1.2	27.4	
12	327	58	0.4	17.7	
13	863	52	0.4	6.0	
14	421	45	0.3	10.7	
15	749	43	0.3	5.7	
16	635	18	0.1	2.8	
17	231	13	0.1	5.6	
**Year**					
2013	3,234	1,318	10.0	40.8	< 0.001
2014	3,706	1,533	11.6	41.4	
2015	4,614	1,429	10.8	31.0	
2016	5,358	2,063	15.7	38.5	
2017	6,048	2,343	17.8	38.7	
2018	5,606	2,209	16.8	39.4	
2019	5,697	2,277	17.3	40.0	
**Sex/gender^b^ and sexual orientation **					
Women	5,870	1,612	12.2	27.5	< 0.001
Heterosexual men	3,631	1,809	13.7	49.8	
MSM	24,762	9,751	74.0	39.4	
*Homosexual*	*22,663*	*9,004*	*92.3^c^*	*39.7*	
*Bisexual*	*2,099*	*747*	*7.7^c^*	*35.6*	
**Origin of culture isolate**					
Urogenital	UN	6,726	51.1	NA	NA
Anorectal	UN	4,889	37.1	NA	
Pharyngeal	UN	1,431	10.9	NA	
Missing	UN	126	1.0	NA	
**Age in years**					
< 25	10,289	3,964	30.1	38.5	0.837
≥ 25	23,974	9,208	69.9	38.4	
**Migration background from an STI/HIV endemic area**					
No	22,728	8,168	62.0	35.9	< 0.001
Yes	11,434	4,984	37.8	43.6	
Missing	101	20	0.2	19.8	
**Number of partners (past 6** **months)**					
0–3	11,431	4,446	33.8	38.9	0.794
4–9	10,494	4,113	31.2	39.2	
≥ 10	11,416	4,489	34.1	39.3	
Missing	922	124	0.9	13.4	
**HIV positive **					
No	28,794	10,828	82.2	37.6	< 0.001
Yes	5,175	2,293	17.4	44.3	
Missing	294	51	0.4	17.3	
**Previous STI (chlamydia, gonorrhoea, syphilis in past year) **					
No	21,460	7,843	59.5	36.5	< 0.001
Yes	11,402	4,876	37.0	42.8	
Missing	1,401	453	3.4	32.3	
**Notified for STI by (ex)partner **					
No	23,553	9,537	72.4	40.5	< 0.001
Yes	10,660	3,625	27.5	34.0	
Missing	50	10	0.1	20.0	
**Reported STI symptoms **					
No	19,363	6,047	45.9	31.2	< 0.001
Yes	14,837	7,116	54.0	48.0	
Missing	63	9	0.1	14.3	

GRAS: Gonococcal Resistance to Antibiotics Surveillance; MSM: men who have sex with men; NA: not applicable; SHC: sexual health centre; STI: sexually transmitted infection; UN: data unavailable.

^a^ SHC participating in GRAS send a sample for culture in the laboratory from all individuals with gonorrhoea symptoms or individuals who tested positive for gonorrhoea with nucleic acid amplification test. If the culture is positive, susceptibility testing is performed.

^b^ Only cis-gender persons were included in the analyses.

^c^ Proportion (%) with antimicrobial susceptibility testing results among total MSM with such results.

### Trends in antimicrobial resistance and decreased susceptibility

**Figure 1:**
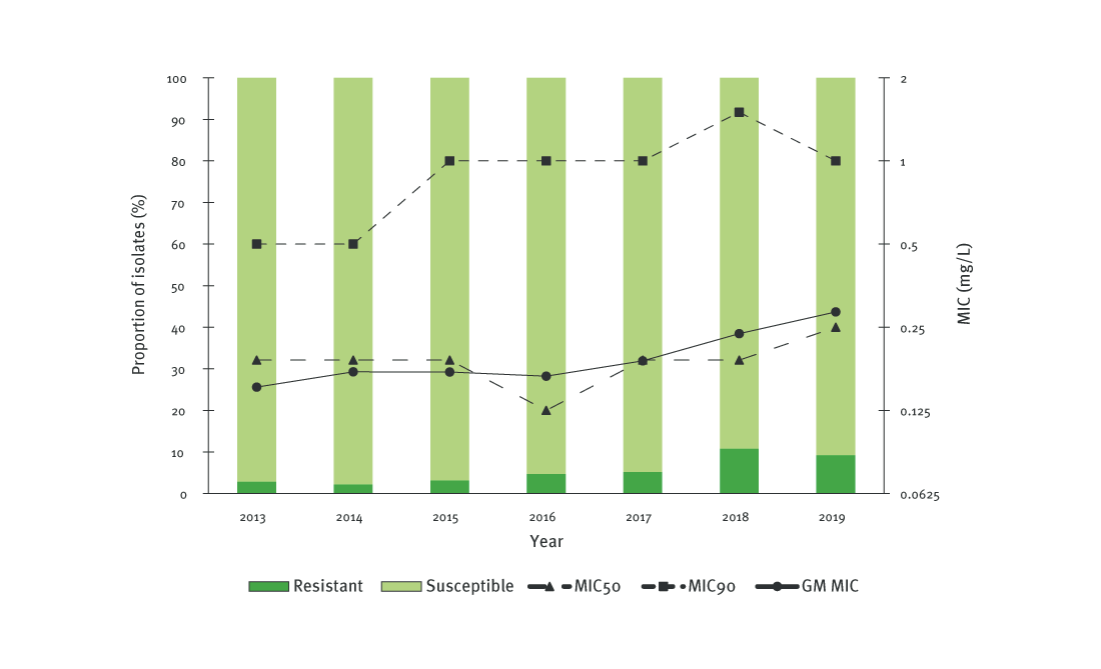
Distribution over time of the proportion of isolates with azithromycin resistance, as well as MIC_50_, MIC_90_ and geometric mean MIC values for azithromycin in GRAS, the Netherlands, 2013–2019 (n = 13,096 isolates^a^)

GM: geometric mean; GRAS: Gonococcal Resistance to Antibiotics Surveillance; MIC: minimum inhibitory concentration.

^a^ For 76 isolates, azithromycin testing results were missing.

**Figure 2:**
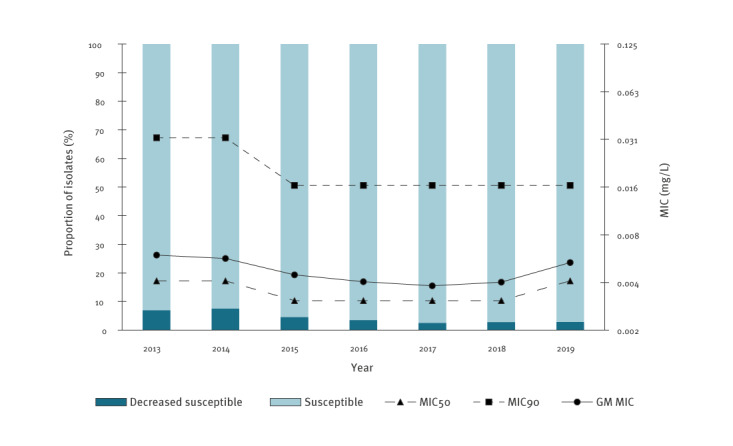
Distribution over time of the proportion of isolates with decreased susceptibility to ceftriaxone, as well as MIC_50_, MIC_90_ and geometric mean MIC values for ceftriaxone in GRAS, the Netherlands, 2013–2019 (n = 13,123^a^)

GM: geometric mean; GRAS: Gonococcal Resistance to Antibiotics Surveillance; MIC: minimum inhibitory concentration.

^a^ For 49 isolates, ceftriaxone testing results were missing.

Resistance to azithromycin appeared to increase during the study period, with 2.8% (37/1,304) of isolates found resistant in 2013 and 9.3% (210/2,264) in 2019 (Figure 1). The MIC_90_ went from 0.5 mg/L to 1 mg/L and the geometric mean MIC from 0.152 mg/L to 0.284 mg/L, whereas the MIC_50_ remained mostly stable around 0.19 mg/L. For ceftriaxone, no resistance has yet been reported in the Netherlands. The proportion of isolates showing decreased susceptibility to ceftriaxone was 7.0% (91/1,306) in 2013 and seemed to have diminished in 2019, when 2.9% (65/2,276) of such isolates were identified (Figure 2). MIC_50_, MIC_90_ and geometric mean MIC values also appeared to decrease after 2013, but the MIC_50_ and geometric mean MIC seemed to slightly increase again in 2019 compared with 2018 to 0.004 mg/L and 0.005 mg/L, respectively. Separate figures for MSM and heterosexuals are shown in Supplementary Figure S2 and S3. In general, levels of resistance/decreased susceptibility were higher among MSM, but tendencies were similar.

### Regional differences in antimicrobial susceptibility

**Figure 3:**
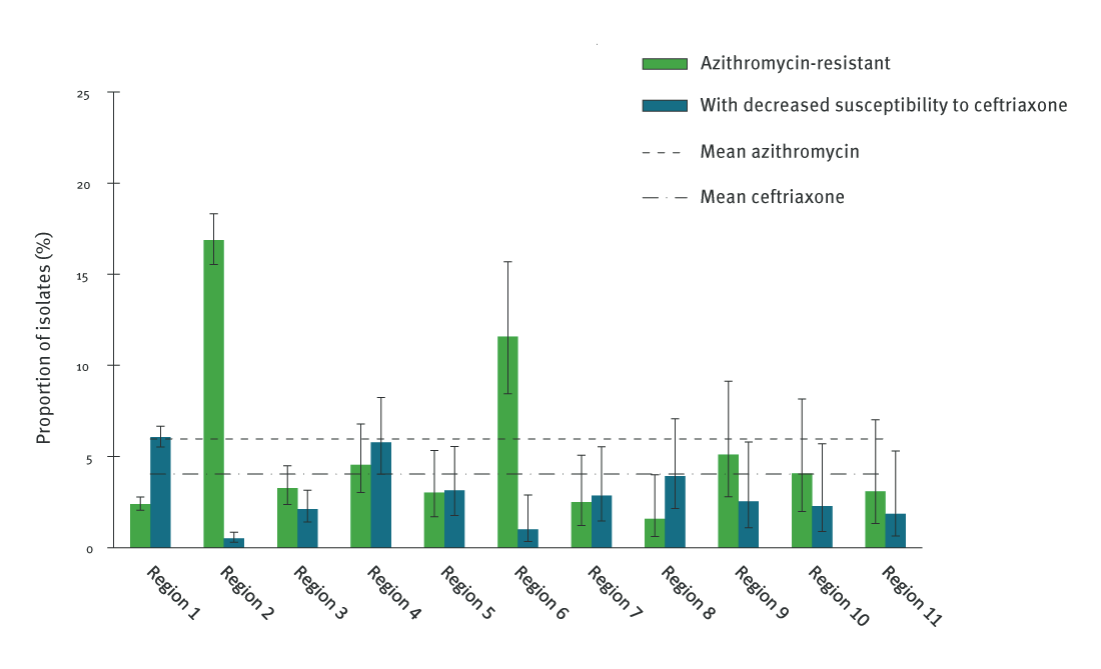
Percentage of isolates resistant to azithromycin and with decreased susceptibility to ceftriaxone per SHC participating in GRAS, the Netherlands, 2013–2019 (n = 13,172)

The percentage of isolates resistant/reduced susceptible was calculated per SHC region for the period of 2013–2019 in total (Figure 3). For azithromycin, the percentage resistant ranged from 0.0% to 16.9%. In the regions with less than 60 isolates (regions 12–17) there was a large uncertainty around the per cent-resistant, and such regions are therefore not included in the figure. But also among the regions with larger numbers the percentages differed substantially. For nine of 11 regions included, the percentage of isolates resistant to azithromycin was lower than the national mean of 6.0%. For ceftriaxone, the percentage of isolates with decreased susceptibility ranged from 0.0% to 6.1%, slightly less than with azithromycin. Considerable regional variability was still observed, also among the regions with more consultations (regions 1‒11). Furthermore, regions with high levels of azithromycin resistance did not necessarily also have high levels of decreased susceptibility to ceftriaxone. For example, region 2 had the highest level of azithromycin resistance (16.9%) and one of the lowest levels of decreased susceptibility to ceftriaxone (0.5%). The geographical location and exact percentages resistance of the regions, including regions 12–17, are shown in Supplementary Table S1.

### Predictors of antimicrobial resistance or decreased susceptibility

In univariable analysis, azithromycin resistance among MSM was associated with age, migration background, number of partners, HIV status, culture origin, reporting STI symptoms, year of consultation and STI testing rate. In multivariable analysis only pharyngeal culture (adjusted odds ratio (aOR): 1.77; 95% CI: 1.38–2.28) and year of consultation (aOR: 1.42; 95% CI: 1.04–1.94 for 2015–2017 and aOR: 3.26; 95% CI: 2.40–4.42 for 2018–2019) remained significantly associated with azithromycin resistance (Table 2). Among women and heterosexual men, ≥ 10 partners, pharyngeal culture and consultation in 2018–2019 were the only variables significantly associated with azithromycin resistance in both univariable and multivariable analysis, with aORs of 1.90 (95% CI: 1.07–3.39), 2.90 (95% CI: 1.58–5.32), and 2.73 (95% CI: 1.50–4.97), respectively (Table 2).

**Table 2:** Predictors of azithromycin resistance in GRAS, the Netherlands, 2013–2019 (n = 13,096^a^)

Characteristic	MSM				Women^b^ and heterosexual men			
	OR	95% CI	aOR^c^	95% CI	OR	95% CI	aOR^c^	95% CI
**Age**								
< 25	Ref.	Ref.	NS	NS	Ref.	Ref.	NS	NS
≥ 25	0.85	0.70–1.02	NS	NS	1.30	0.87–1.96	NS	NS
**Sex**								
Female	NA	NA	NA	NA	Ref.	Ref.	NS	NS
Male	NA	NA	NA	NA	0.73	0.48–1.10	NS	NS
**Sexual orientation**								
Homosexual	Ref.	Ref.	NS	NS	NA^b^	NA^b^	NA^b^	NA^b^
Bisexual	1.10	0.83–1.46	NS	NS	NA^b^	NA^b^	NA^b^	NA^b^
**STI/HIV endemic migration background**								
No	Ref.	Ref.	NS	NS	Ref.	Ref.	NS	NS
Yes	1.21	1.03–1.43	NS	NS	0.81	0.54–1.23	NS	NS
**Number of partners (past 6** **months)**								
0–3	Ref.	Ref.	NS	NS	Ref.	Ref.	Ref.	Ref.
4–9	0.86	0.70–1.05	NS	NS	0.86	0.50–1.47	1.03	0.59–1.78
≥ 10	0.80	0.66–0.98	NS	NS	1.62	0.93–2.82	1.90	1.07–3.39
**HIV status **								
Negative	Ref.	Ref.	NS	NS	NA^b^	NA^b^	NA^b^	NA^b^
Positive	0.67	0.55–0.82	NS	NS	NA^b^	NA^b^	NA^b^	NA^b^
**Origin of culture isolate**								
Urogenital	Ref.	Ref.	Ref.	Ref.	Ref.	Ref.	Ref.	Ref.
Anorectal	1.07	0.90–1.27	1.02	0.84–1.23	1.38	0.68–2.80	1.08	0.48–2.46
Pharyngeal	1.66	1.32–2.08	1.77	1.38–2.28	3.15	1.79–5.53	2.90	1.58–5.32
**Previous STI (chlamydia, gonorrhoea, syphilis in past year)**								
No	Ref.	Ref.	NS	NS	Ref.	Ref.	NS	NS
Yes	0.96	0.82–1.12	NS	NS	1.26	0.79–2.02	NS	NS
**Notified for STI by (ex)partner**								
No	Ref.	Ref.	NS	NS	Ref.	Ref.	NS	NS
Yes	0.95	0.80–1.14	NS	NS	0.82	0.51–1.34	NS	NS
**Reported STI symptoms**								
No	Ref.	Ref.	NS	NS	Ref.	Ref.	NS	NS
Yes	0.84	0.72–0.98	NS	NS	1.02	0.66–1.56	NS	NS
**Year of consultation**								
2013–2014	Ref.	Ref.	Ref.	Ref.	Ref.	Ref.	Ref.	Ref.
2015–2017	1.91	1.41–2.57	1.42	1.04–1.94	1.03	0.55–1.95	0.82	0.42–1.60
2018–2019	4.14	3.11–5.53	3.26	2.40–4.42	3.59	2.04–6.32	2.73	1.50–4.97
**STI testing rate (regional)**								
Low	Ref.	Ref.	NS	NS	Ref.	Ref.	NS	NS
Middle	0.72	0.39–1.30	NS	NS	1.70	0.31–9.38	NS	NS
High	1.59	1.09–2.31	NS	NS	3.19	0.78–13.05	NS	NS

aOR: adjusted odds ratio; CI: confidence interval; GRAS: Gonococcal Resistance to Antibiotics Surveillance; MSM: men who have sex with men; NA: not applicable; NS: not significant; OR: odds ratios; Ref.: reference category; SHC: sexual health centre; STI: sexually transmitted infection.

^a^ For 76 isolates, azithromycin testing results were missing.

^b^ All women were grouped together, regardless of the sex of their sexual partners. HIV status was not included in the women/heterosexual men models due to low numbers of HIV infections in this group.

^c^ Also adjusted for SHC region by random intercept.

For ceftriaxone, among MSM, decreased susceptibility was associated with number of partners, culture origin, year of consultation and STI testing rate in univariable analysis, of which pharyngeal culture (aOR: 1.87; 95% CI: 1.40–2.48) year of consultation (aOR: 0.39; 95% CI: 0.31–0.49 for 2015–2017 and aOR: 0.35; 95% CI: 0.27–0.45 for 2018–2019) remained significant in the multivariable model (Table 3). Among women and heterosexual men, high number of partners (aOR: 1.88; 95% CI: 1.05–3.37 for 4–9 partners and aOR: 4.94;  95% CI: 2.87–8.52 for ≥ 10 partners), anorectal and pharyngeal culture (aOR: 3.11; 95% CI: 1.66–5.83 and aOR: 3.22; 95% CI: 1.64–6.28 respectively) and year of consultation (aOR: 0.44; 95% CI: 0.27–0.75 for 2015–2017 and aOR: 0.33; 95% CI: 0.17–0.63 for 2018–2019) were significantly associated with decreased susceptibility in multivariable analysis (Table 3).

**Table 3:** Predictors of ceftriaxone reduced susceptibility in GRAS, the Netherlands, 2013–2019 (n = 13,123^a^)

Characteristic	MSM				Women^b^ and heterosexual men			
	OR	95% CI	aOR^c^	95% CI	OR	95% CI	aOR^c^	95% CI
**Age**								
< 25	Ref.	Ref.	NS	NS	Ref.	Ref.	NS	NS
≥ 25	0.95	0.75–1.20	NS	NS	1.52	1.00–2.32	NS	NS
**Sex**								
Female	NA	NA	NA	NA	Ref.	Ref.	NS	NS
Male	NA	NA	NA	NA	0.50	0.32–0.78	NS	NS
**Sexual preference**								
Homosexual	Ref.	Ref.	NS	NS	NA^b^	NA^b^	NA^b^	NA^b^
Bisexual	0.84	0.57–1.23	NS	NS	NA^b^	NA^b^	NA^b^	NA^b^
**STI/HIV endemic migration background**								
No	Ref.	Ref.	NS	NS	Ref.	Ref.	NS	NS
Yes	0.98	0.79–1.21	NS	NS	0.82	0.53–1.26	NS	NS
**Number of partners (past 6** **months)**								
0–3	Ref.	Ref.	NS	NS	Ref.	Ref.	Ref.	Ref.
4–9	1.23	0.94–1.61	NS	NS	1.99	1.13–3.52	1.88	1.05–3.37
≥ 10	1.3	1.00–1.68	NS	NS	6.96	4.24–11.42	4.94	2.87–8.52
**HIV status**								
Negative	Ref.	Ref.	NS	NS	NA	NA	NA	NA
Positive	0.86	0.68–1.09	NS	NS	NA	NA	NA	NA
**Origin of culture isolate**								
Urogenital	Ref.	Ref.	Ref.	Ref.	Ref.	Ref.	Ref.	Ref.
Anorectal	0.93	0.75–1.14	1.14	0.91–1.42	3.38	1.93–5.92	3.11	1.66–5.83
Pharyngeal	1.53	1.17–2.01	1.87	1.40–2.48	3.87	2.18–6.87	3.22	1.64–6.28
**Previous STI (chlamydia, gonorrhoea, syphilis in past year)**								
No	Ref.	Ref.	NS	NS	Ref.	Ref.	NS	NS
Yes	0.91	0.75–1.11	NS	NS	0.96	0.57–1.63	NS	NS
**Notified for STI by (ex)partner**								
No	Ref.	Ref.	NS	NS	Ref.	Ref.	NS	NS
Yes	1.08	0.88–1.34	NS	NS	0.86	0.52–1.41	NS	NS
**Reported STI symptoms**								
No	Ref.	Ref.	NS	NS	Ref.	Ref.	NS	NS
Yes	0.86	0.71–1.04	NS	NS	0.66	0.43–1.00	NS	NS
**Year of consultation**								
2013–2014	Ref.	Ref.	Ref.	Ref.	Ref.	Ref.	Ref.	Ref.
2015–2017	0.39	0.31–0.49	0.39	0.31–0.49	0.58	0.37–0.93	0.44	0.27–0.75
2018–2019	0.34	0.26–0.43	0.35	0.27–0.45	0.35	0.19–0.66	0.33	0.17–0.63
**STI testing rate (regional) **								
Low	Ref.	Ref.	NS	NS	Ref.	Ref.	NS	NS
Middle	1.33	0.66–2.68	NS	NS	1.09	0.37–3.21	NS	NS
High	2.01	1.19–3.39	NS	NS	0.85	0.37–1.97	NS	NS

aOR: adjusted odds ratio; CI: confidence interval; GRAS: Gonococcal Resistance to Antibiotics Surveillance; MSM: men who have sex with men; NA: not applicable; NS: not significant; OR: odds ratios; Ref.: reference category; STI: sexually transmitted infection.

^a^ For 49 isolates, ceftriaxone testing results were missing.

^b^ All women were grouped together, regardless of the sex of their sexual partners. HIV status was not included in the women/heterosexual men models due to low numbers of HIV infections in this group.

^c^ Also adjusted for SHC region by random intercept.

### Regional variance and population characteristics

Regional variance of azithromycin resistance in the empty model was 0.450 and increased to 0.513 after addition of population characteristic variables, resulting in a PCV of −14.02%. Previous STI contributed most to the change in variance, with −2.72%. Variables that decreased regional variance after addition were migration background, being notified for STI by a partner and year of consultation (Table 4). For ceftriaxone, regional variance was 0.444 in the empty model and decreased to 0.415 in the extended model, with a PCV of 6.51%. Year of consultation, number of partners and origin of culture isolate contributed the most to decreasing regional variance. HIV status resulted in the largest increase (−4.42%) (Table 4).

**Table 4:** Regional variance models of azithromycin resistance and ceftriaxone reduced susceptibility, the Netherlands, 2013–2019 (n = 13,172)

Model description	Azithromycin			Ceftriaxone		
	Regional variance	95% CI	PCV (%)	Regional variance	95% CI	PCV (%)
Empty model	0.450	0.195–1.039	NA	0.444	0.181–1.087	NA
Model with all characteristics	0.513	0.226–1.162	−14.02	0.415	0.160–1.074	6.51
Variables	Regional variance in model 1−k	Contribution to variance (%)		Regional variance in model 1−k	Contribution to variance (%)	
Age	0.511	−0.48		0.415	−0.01	
Sex and sexual orientation	0.501	−2.32		0.422	1.63	
STI/HIV endemic migration background	0.514	0.10		0.409	−1.53	
Number of partners	0.501	−2.35		0.426	2.45	
HIV status	0.513	−0.03		0.398	−4.42	
Origin of culture isolate	0.503	−1.93		0.425	2.24	
Previous STI	0.499	−2.72		0.422	1.56	
Notified for STI by (ex)partner	0.514	0.20		0.416	0.19	
Symptoms	0.513	−0.02		0.412	−0.79	
Year of consultation	0.526	2.41		0.435	4.66	

NA: not applicable; PCV: proportional change in variance; STI: sexually transmitted infection.

## Discussion

In this study of the Dutch national gonococcal surveillance programme (GRAS) we did not observe resistance to ceftriaxone and more recent isolates in GRAS generally had lower MICs than in earlier years. Between 2013 and 2019, annual prevalence of isolates with azithromycin resistance appeared to increase, as did geometric mean MIC and MIC_90_ values. These results concur with those of the multivariable regression analyses, where significantly increased risk for azithromycin resistance was found in the more recent years while this effect was reversed for ceftriaxone. The regression analyses also showed an association of strains of pharyngeal origin and high number of partners with azithromycin resistance and decreased susceptibility to ceftriaxone. Regional variance could not be explained by population characteristics.

The apparent increasing azithromycin resistance reflects trends seen in other gonococcal surveillance systems worldwide [13-16]. While the WHO and ECDC recommend azithromycin/ceftriaxone dual therapy [6,7], in the Netherlands, ceftriaxone is used as monotherapy for gonorrhoea treatment since 2006. However, co-infection of gonorrhoea and chlamydia is common among SHC visitors; in 2019, 42% of heterosexuals and 23% of MSM diagnosed with gonorrhoea also had a chlamydia infection and were therefore treated with azithromycin in addition to ceftriaxone [1]. Azithromycin may also have been given when clinical treatment of urethritis (when symptoms were not indicating gonorrhoea) was applied before receiving the gonorrhoea testing results. This could partially explain why azithromycin resistance is also developing in the Netherlands.

The ceftriaxone trends observed in the Netherlands are difficult to compare with other countries. Similar results, with no evidence of decreasing susceptibility for ceftriaxone and only incidental resistant cases are described in surveillance reports with data up to 2019 from the European, United States (US), and Australian gonococcal resistance surveillance [13,15,16]. However, in the Asia Pacific region taken as a whole, increasingly high levels of ceftriaxone resistance were seen from 2011 to 2016, with multiple countries reporting levels over 5% [14]. Additionally, in the United Kingdom (UK), the gonococcal resistance surveillance programme showed a clear MIC drift towards decreased susceptibility to ceftriaxone between 2014 and 2018, a concerning signal, though this trend halted in 2019 [17]. Molecular epidemiology of decreased susceptibility to ceftriaxone and resistance to azithromycin have been studied in more detail in the Amsterdam region (SHC region 1), providing 52% of all strains included in this study. Resistance and decreased susceptibility were restricted to certain clonal strain populations which appeared and disappeared over time [18-20]. Further study on strains from other regions would be required to assess whether a nationwide spread of clonal strain populations is related to the observed trends in resistance and decreased susceptibility.

A big strength of the GRAS programme is the inclusion of microbial susceptibility data within the national STI surveillance, allowing for many individual- and sexual behaviour characteristics to be included in the analyses. Our regression analyses showed that pharyngeal origin of strains was significantly associated with AMR and decreased susceptibility for both azithromycin and ceftriaxone, and among both MSM and heterosexuals. Pharyngeal gonorrhoea infections are often associated with transmission and AMR development [21,22]. First, because oropharyngeal infections are mainly asymptomatic and therefore can act as a reservoir of sustained transmission. Second, *N. gonorrhoeae* present in the pharynx could acquire molecular AMR elements (e.g. by homologous recombination) from commonly present non-gonococcal *Neisseria* species. And last, pharyngeal gonococci are often exposed to lower antimicrobial concentrations due to the suboptimal pharmacodynamic and pharmacokinetic properties of many antimicrobials in the pharynx [21,22]. However, in the GRAS programme, pharyngeal infections prove difficult to culture and yield positive results less often than urogenital and anorectal material (20% positive vs 70% and 55% in 2019 respectively). Also, in this study only one anatomical location per patient could be included. This could have introduced some bias in our results, for example if AMR influences strain fitness or if there are other yet unidentified risk factors associated with both pharyngeal infection and resistance. Future studies using within-person comparison of gonococcal susceptibility patterns of multiple anatomical locations could provide more insight into the role of pharyngeal infections in development and transmission of resistant gonorrhoea.

High number of partners was also associated with azithromycin resistance and decreased susceptibility to ceftriaxone, but only among heterosexuals. This was not commonly reported by other studies [23]. It might be indicative of higher risk behaviour contributing to AMR development or transmission. However, none of the other included variables were significantly associated with AMR, even though most of these are also related to STI risk and risk behaviour. Therefore, the cause of this association remains unclear.

For the first time, regional differences within the GRAS national surveillance data were reported. For both azithromycin and ceftriaxone, the percentage of isolates showing resistance/decreased susceptibility differed between SHC regions. Especially for azithromycin, most regions had a resistance level that was lower than the national average. The model quantifying regional variance showed that correction for population characteristics barely explained or even increased regional variance. It is possible that there are unmeasured or residual confounding factors that explain these regional differences. For example, there are regional differences in testing practices and laboratory methods since participating laboratories are free to choose which Etest and culture plates they use. Almost all laboratories use the Biomerieux Etest, but the culture plates that are used differ greatly. However, external quality assessments of the participating laboratories showed no major differences in MICs between laboratories, with > 80% of reported MIC values being within one dilution difference of the correct value of the reference strains. Hence, we do not expect laboratory methodology to be the sole explanation for the found regional differences. Therefore, we suggest that there are regional differences in gonorrhoea AMR in the Netherlands. Previous studies from the UK have found clustering of specific gonorrhoea strains geographically, within sexual networks, and within ‘core high-risk groups’, even when only looking at the London area [24,25]. Additionally, Town et. al. showed independent emergence of AMR within different gonorrhoea strains in separate sexual networks in England [26]. So, differences found in the Netherlands could be due to regional clustering of gonorrhoea strains with different antimicrobial susceptibility. These findings underline the importance of having a national surveillance network with wide coverage, both geographically and in terms of included population ‘core’ groups. Reporting findings also on a regional level may help signal AMR emergence in a timely manner and inform region specific interventions to limit AMR transmission.

This study has several limitations. First, since susceptibility data of many patients with gonorrhoea are not available due to lack of (successful) cultures, numbers in our analyses were relatively low, especially among heterosexuals. Therefore, some characteristics could not be included in our analyses (sex work) or had to be included with limited level of detail (year of consultation, migration background), which might have caused us to miss certain predictors of resistance. For example, we did not find any association with migration background, while this has been shown in other studies that looked at specific countries or areas of birth [23,27,28]. Second, we did not have any data available on previous antibiotic use or on background usage by region, which might also be risk factors for gonococcal resistance due to selective pressure [23,29]. We did include having a previous STI diagnosis in our model, which is linked to previous STI treatment, but no significant association with AMR was found. Third, the clinical relevance of the chosen outcome measures is limited. For azithromycin, only an epidemiological cut-off is available to determine AMR and for ceftriaxone we used decreased susceptibility in the absence of resistance. The clinical relevance of both with respect to treatment failure is unknown. Furthermore, azithromycin is not used for treatment of gonorrhoea in the Netherlands, moreover the updated UK and US guidelines have recommended ceftriaxone monotherapy in 2018 and 2020, respectively [30,31]. However, we did find the same risk factors for azithromycin resistance and decreased susceptibility to ceftriaxone, indicating that there might be similar groups or mechanisms involved in the development and/or transmission of resistance to both antimicrobials. Last, molecular typing is not routinely performed in GRAS and therefore genotype data were not available to include in our analyses. Future studies using molecular GRAS data could give more insight into whether our observed regional differences are caused by geographical clustering of different strains.

## Conclusions and recommendations

In conclusion, data from the Dutch gonococcal AMR surveillance show no resistance or decreasing susceptibility for ceftriaxone but suggest increasing azithromycin resistance. Regional differences were observed in both levels of ceftriaxone and azithromycin susceptibility, which could not be explained by population characteristics. Including molecular data in gonococcal surveillance may give more insight into geographical clustering of different strains. These results could be used to improve gonococcal AMR surveillance. We recommend surveillance systems to have a broad geographical coverage, and to analyse and/or report their data on a regional level as well. This because sentinel surveillance depending on a limited number of included areas or only looking at trends on a national level might miss early signs of developing resistance. Furthermore, the association of pharyngeal strain origin with resistance/decreased susceptibility underlines the importance of extragenital testing and including especially pharyngeal infections in gonococcal resistance surveillance programmes.

## Supplementary Data

296000 Supplementary Material
