# Supplementary material for: Trends and regional variations of gonococcal antimicrobial resistance in the Netherlands, 2013 to 2019
Source: Euro Surveill. 2022 Aug 25;27(34):2200081. doi: 10.2807/1560-7917.ES.2022.27.34.2200081 (PMC9413857; doi:10.2807/1560-7917.ES.2022.27.34.2200081)

# Trends and regional variations of gonococcal antimicrobial resistance in the Netherlands, 2013 to 2019: supplementary file

This supplementary material is hosted by Eurosurveillance as supporting information alongside the article Trends and regional variations of gonococcal antimicrobial resistance in the Netherlands, 2013 to 2019 on behalf of the authors, who remain responsible for the accuracy and appropriateness of the content. The same standards for ethics, copyright, attributions and permissions as for the article apply. Supplements are not edited by Eurosurveillance and the journal is not responsible for the maintenance of any links or email addresses provided therein.

**Table S1** Population, STI testing, GRAS participation and gonococcal resistance characteristics per SHC region included in GRAS

| Region* | | N inhabitants age 15-65 in 2019 | N persons with at least 1 SHC consultation age 15-65 in 2019 | STI testing rate at SHC per 1,000 inhabitants in 2019 | N isolates included in GRAS 2013-2019 | % azithromycin resistant 2013-2019 % (95% CI) | % ceftriaxone decreased susceptible 2013-2019 % (95% CI) |
|---|---|---|---|---|---|---|---|
| GGD Amsterdam | 1 | 755,121 | 50,299 | 66.6 | 6815 | 2.4 (2.0-2.8) | 6.1 (5.5-6.7) |
| GGD Rotterdam Rijnmond | 2 | 866,272 | 12,933 | 14.9 | 2802 | 16.9 (15.5-18.3) | 0.5 (0.3-0.8) |
| GGD Haaglanden | 3 | 733,951 | 11,825 | 16.1 | 1103 | 3.3 (2.4-4.5) | 2.1 (1.4-3.1) |
| GGD Zuid Limburg | 4 | 380,851 | 6,551 | 17.2 | 484 | 4.5 (3.0-6.8) | 5.8 (4.0-8.2) |
| GGD regio Utrecht | 5 | 883,974 | 4,311 | 4.9 | 364 | 3.0 (1.7-5.3) | 3.2 (1.8-5.6) |
| GGD Gelderland-Zuid | 6 | 369,432 | 5,854 | 15.8 | 302 | 11.6 (8.5-15.7) | 1.0 (0.3-2.9) |
| GGD Regio Twente | 7 | 401,960 | 4,967 | 12.4 | 280 | 2.5 (1.2-5.1) | 2.9 (1.5-5.5) |
| GGD West Brabant | 8 | 452,908 | 4,394 | 9.7 | 256 | 1.6 (0.6-4.0) | 3.9 (2.1-7.1) |
| GGD Hart voor Brabant | 9 | 691,669 | 4,114 | 5.9 | 199 | 5.1 (2.8-9.1) | 2.5 (1.1-5.8) |
| GGD Hollands Noorden | 10 | 413,769 | 4,488 | 10.8 | 176 | 4.1 (2.0-8.2) | 2.3 (0.9-5.7) |
| GGD Limburg-Noord | 11 | 329,134 | 2,040 | 6.2 | 162 | 3.1 (1.3-7.0) | 1.9 (0.6-5.3) |
| Dienst Gezondheid & Jeugd Zuid-Holland Zuid | 12 | 288,515 | 1,569 | 5.4 | 58 | 1.8 (0.3-9.3) | 1.9 (0.3-9.8) |
| GGD Brabant Zuid-Oost | 13 | 503,643 | 4,361 | 8.7 | 52 | 8.5 (3.4-19.9) | 5.8 (2.0-15.6) |
| GGD Fryslân | 14 | 406,600 | 1,720 | 4.2 | 45 | 11.1 (4.4-25.3) | 2.2 (0.4-11.6) |
| GGD Kennemerland | 15 | 351,904 | 4,578 | 13.0 | 43 | 4.8 (1.3-15.8) | 7.0 (2.4-18.6) |
| GGD Groningen | 16 | 387,654 | 3,486 | 9.0 | 18 | 0.0 (0.0-39.0) | 0.0 (0.0-18.4) |
| GGD Zeeland | 17 | 235,058 | 1,006 | 4.3 | 13 | 7.7 (1.4-33.3) | 0.0 (0.0-22.8) |

**Figure S1: Geographical location of SHC regions* participating in GRAS**

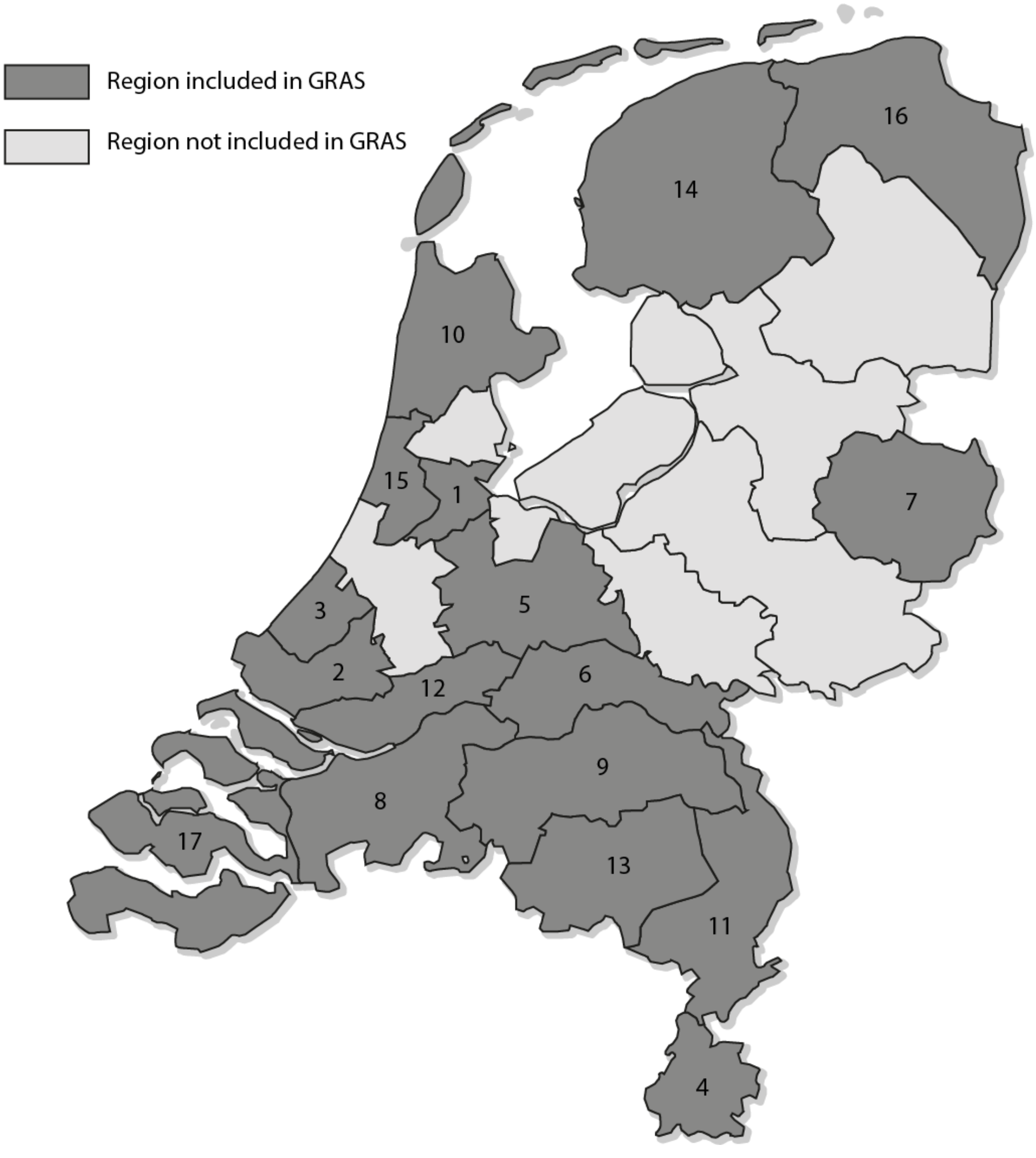


*region numbers correspond to numbers in table 1, table S1 and figure 3

**Figure S2A: Trends in percentage resistance, $MIC_{50}$, $MIC_{90}$ and geometric mean (GM) MIC values for azithromycin among men who have sex with men in GRAS, 2013-2019**

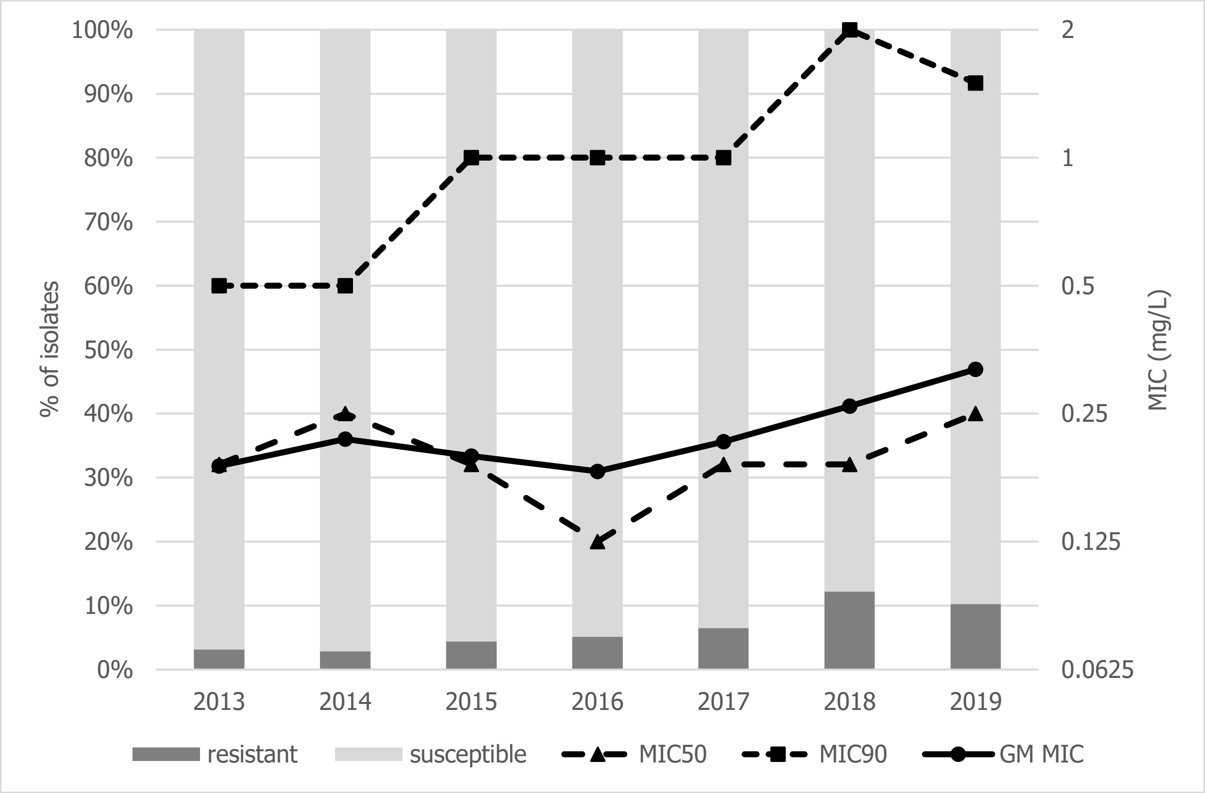


**Figure S2B: Trends in percentage resistance, MIC50, MIC90 and geometric mean (GM) MIC values for azithromycin among women and heterosexual men in GRAS, 2013-2019**

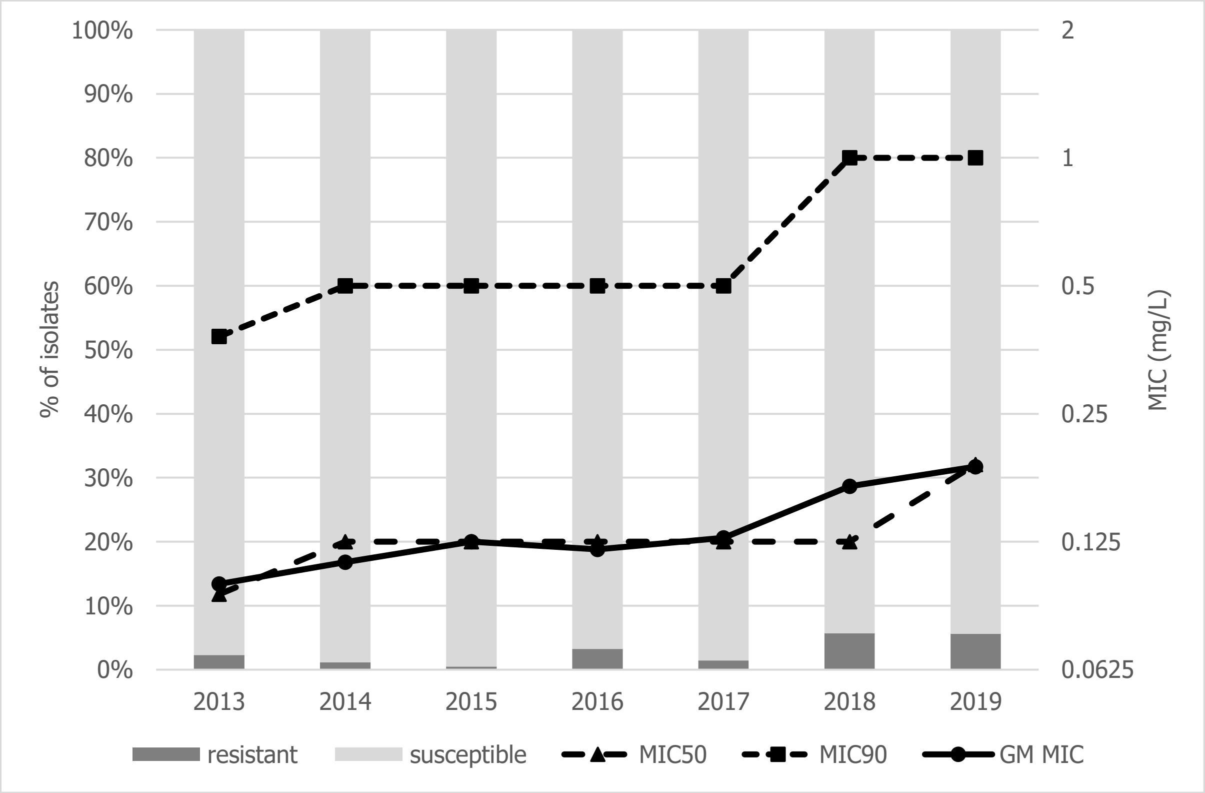

**Figure S3A: Trends in percentage decreased susceptibility, MIC50, MIC90 and geometric mean (GM) MIC values for ceftriaxone among men who have sex with men in GRAS, 2013-2019**

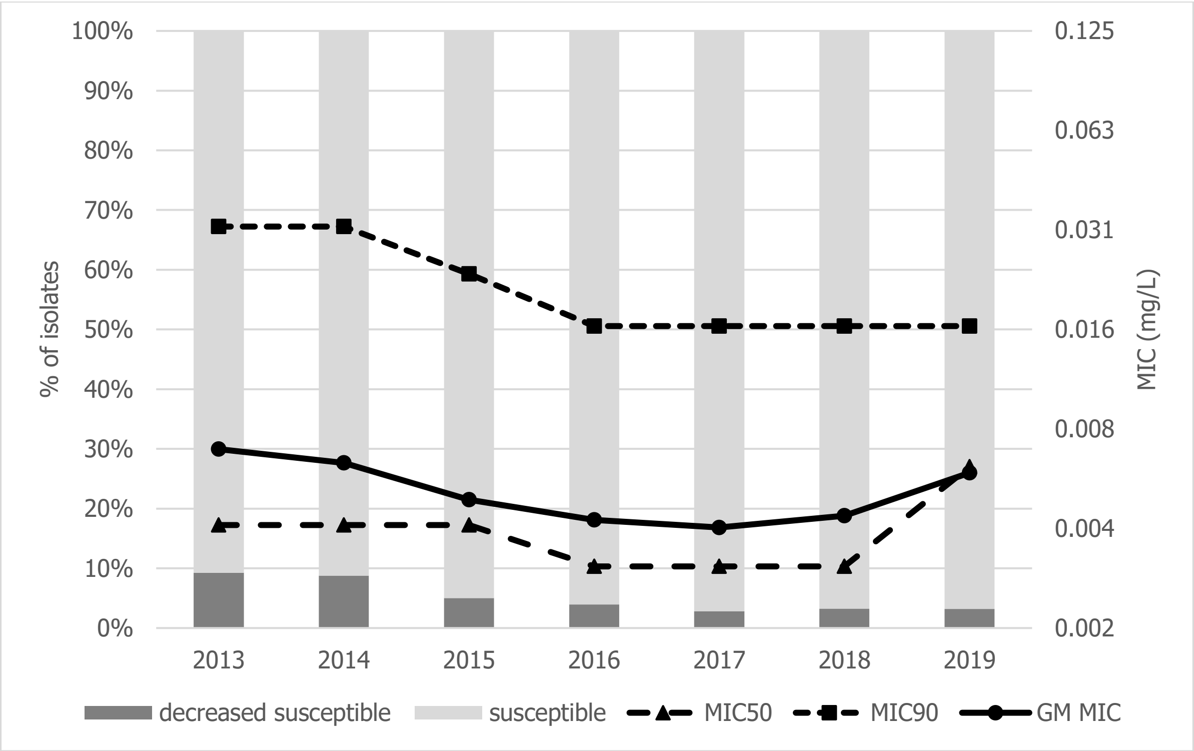


**Figure S3B: Trends in percentage decreased susceptibility, $MIC_{50}$, $MIC_{90}$ and geometric mean (GM) MIC values for ceftriaxone among women and heterosexual men in GRAS, 2013-2019**

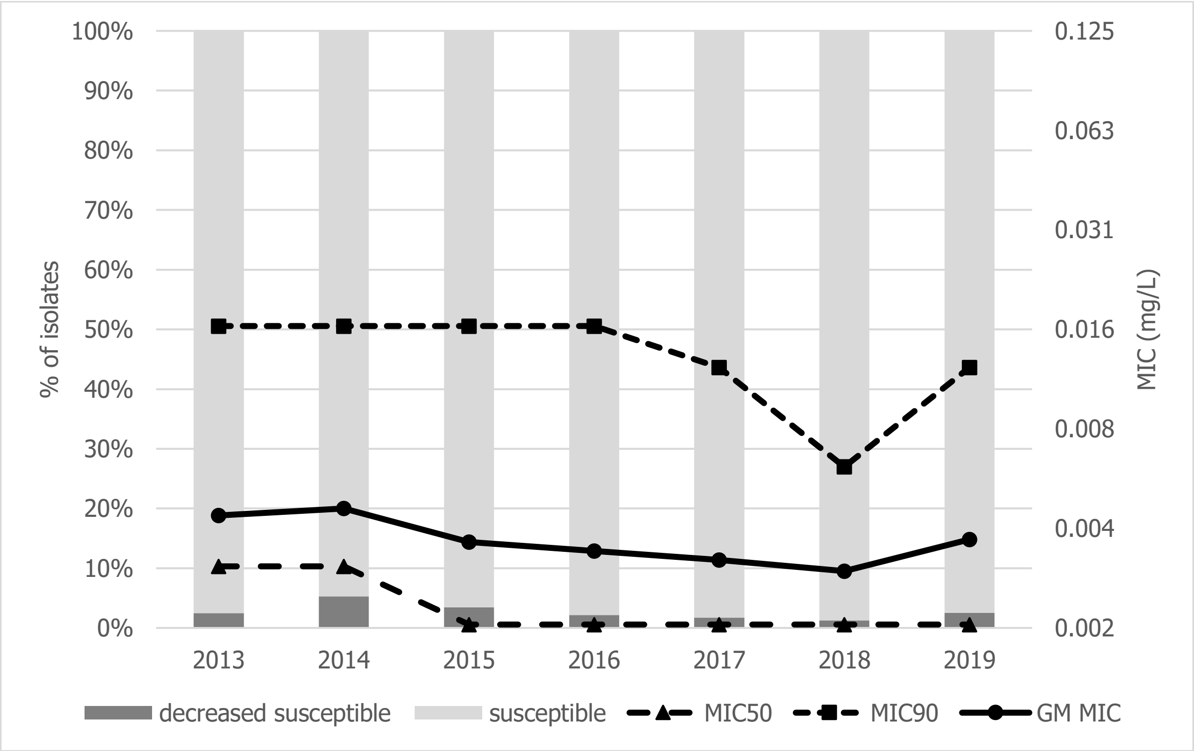

Supplement: Supplementary Material [file 2200081_SupplementaryMaterial.pdf]
